# *Grxcr1* regulates hair bundle morphogenesis and is required for normal mechanoelectrical transduction in mouse cochlear hair cells

**DOI:** 10.1371/journal.pone.0261530

**Published:** 2022-03-02

**Authors:** Beatriz Lorente-Cánovas, Stephanie Eckrich, Morag A. Lewis, Stuart L. Johnson, Walter Marcotti, Karen P. Steel

**Affiliations:** 1 Wolfson Centre for Age-Related Diseases, King’s College London, London, United Kingdom; 2 Wellcome Sanger Institute, Hinxton, United Kingdom; 3 School of Biosciences, University of Sheffield, Sheffield, United Kingdom; 4 Neuroscience Institute, University of Sheffield, Sheffield, United Kingdom; Indiana University School of Medicine, UNITED STATES

## Abstract

Tasmanian devil (*tde*) mice are deaf and exhibit circling behaviour. Sensory hair cells of mutants show disorganised hair bundles with abnormally thin stereocilia. The origin of this mutation is the insertion of a transgene which disrupts expression of the *Grxcr1* (glutaredoxin cysteine rich 1) gene. We report here that *Grxcr1* exons and transcript sequences are not affected by the transgene insertion in *tde* homozygous (*tde/tde*) mice. Furthermore, 5’RACE PCR experiments showed the presence of two different transcripts of the *Grxcr1* gene, expressed in both *tde/tde* and in wild-type controls. However, quantitative analysis of *Grxcr1* transcripts revealed a significantly decreased mRNA level in *tde/tde* mice. The key stereociliary proteins ESPN, MYO7A, EPS8 and PTPRQ were distributed in hair bundles of homozygous *tde* mutants in a similar pattern compared with control mice. We found that the abnormal morphology of the stereociliary bundle was associated with a reduction in the size and Ca^2+^-sensitivity of the mechanoelectrical transducer (MET) current. We propose that GRXCR1 is key for the normal growth of the stereociliary bundle prior to the onset of hearing, and in its absence hair cells are unable to mature into fully functional sensory receptors.

## Introduction

Hair cells are specialized sensory receptors responsible for the conversion of mechanical stimuli into electrical signals, which are relayed to the central auditory system via the afferent nerve fibres. Hair bundles protrude from their apical surfaces. The hair bundle is composed of rows of microvilli-like elements called stereocilia, which are graded in height to form a staircase-like structure [1]. Mechanoelectrical transduction occurs when mechanically-gated ion channels, located at the tips of the stereocilia, open in response to sound induced movement of the hair bundle. Stereocilia are composed of a rigid paracrystalline array of parallel, uniformly polarized and regularly cross-linked actin filaments [2]. The actin filaments are oriented such that the “plus” or “barbed” end point is towards the tip of the stereocilia, and the “minus” or “pointed” end is at the base [3]. In hair cells of the mammalian cochlea, each hair bundle has 3–4 height-ranked rows of stereocilia that are coupled to one another by extracellular links of several types [4], including the tip links that are required for the gating of the mechanoelectrical transducer channels [5, 6]. The length and width of individual stereocilia are remarkably similar, not only within each row of a single hair bundle but also between equivalent rows on adjacent hair cells [2], indicating that the growth and maintenance of the hair bundle structure is likely to be tightly regulated. Indeed, many molecules have been identified as crucial for the normal development and function of the hair bundles by the investigation of mutations causing deafness [1, 7–9]. Despite the identification of several key stereociliary proteins, the mechanisms underlying the elongation and widening of the stereocilia during the early stages of development are still not fully understood.

The *Grxcr1* gene encodes glutaredoxin cysteine rich 1, GRXCR1, which has been shown to target actin-rich structures in cultured cells and is mainly localized at the stereociliary bundles in cochlear and vestibular hair cells [10]. Mutations in the human *GRXCR1* gene cause a form of autosomal-recessive non-syndromic hearing impairment DFNB25 [11], with symptoms ranging from progressive hearing loss to profound deafness [11, 12]. Mice homozygous for mutations in the *Grxcr1* gene exhibit profound deafness and vestibular defects [10, 13, 14]. In these mutant mice, the hair cells show abnormally thin stereocilia and disorganized bundles, a pattern which can be detected from birth and becomes progressively more pronounced with increasing age leading eventually to hair cell degeneration [10, 14]. The Tasmanian devil (*Grxcr1*^*tde*^) mutation has a similar phenotype to pirouette (*Grxcr1*^*pir*^), and the non-complementation of these two mutations when crossed indicated that *pir* and *tde* are allelic [15]. In zebrafish, mutations in the *Grxcr1* gene also leads to hair cells having thin stereocilia, and it has been proposed that GRXCR1 regulates normal stereocilia growth by regulating the interaction between the Usher proteins USH1C and USH1G [16], which are known to be crucial for mechanoelectrical transduction [7]. Although the above studies have highlighted the importance of GRXCR1 for hearing, the functional and molecular consequences of mutations in the *Grxcr1* gene in hair cells are still unknown.

Here, using Tasmanian devil (*tde*) mice, we found that *Grxcr1* mRNA is considerably down-regulated in homozygous *tde* mice although all exon sequences remain intact. Both mutant and wild-type inner ear samples showed two transcripts, with the minor transcript skipping exon 2. The down-regulation of *Grxcr1* did not change the distribution within stereocilia of several key proteins, including ESPN, which is essential for the assembly and stabilization of the actin filaments [17], EPS8, required for normal stereocilia elongation [18], and both the unconventional MYO7A [19, 20] and PTPRQ [21], known to be essential for normal hair bundle morphogenesis. We found that the disrupted hair bundles were still capable of mechanoelectrical transduction when displaced with a fluid jet, but the size and Ca^2+^ sensitivity of the mechanoelectrical transducer (MET) current were largely reduced. Hair cells were also unable to acquire the adult-like basolateral membrane proteins, preventing them from becoming fully functional sensory receptors, which ultimately leads to hair cell loss at later stages [14].

## Materials and methods

### Ethical statement

Mouse studies were carried out in accordance with UK Home Office regulations and the UK Animals (Scientific Procedures) Act of 1986 (ASPA) under UK Home Office licences, and the study was approved by the Sanger Institute, King’s College London and University of Sheffield’s Ethical Review Committees. Mice were culled using cervical dislocation as approved under these licences to minimise any possibility of suffering.

### Tasmanian devil mice

Tasmanian devil (*Grxcr1*^*tde*^, referred to as *tde*) mice arose from microinjection of a human placental alkaline phosphatase genomic construct causing an insertional mutation [22]. The line was maintained on a mixed C57BL/6J; CBA/Ca genetic background. Both male and female mice were used as we observed no differences between them. The colony was generally maintained by homozygote by heterozygote matings, and experiments used homozygous mutants compared with heterozygous or wild type littermates as controls. Mice were group-housed in individually-ventilated cages with lights on a 12 hour on/12 hour off cycle, and experiments were conducted throughout the working day, from two hours to ten hours after lights were switched on. Mice were inspected for any signs of ill-health every day, as required by the UK Home Office, and were maintained in a Specific Pathogen Free facility. Genotypes were determined by abnormal behaviour indicative of a balance defect in mature mice or for mice younger than two weeks old by examination of the other ear by either scanning electron microscopy and detection of thin stereocilia in homozygotes by two independent viewers or by direct imaging in dissected organ of Corti, or by qRT-PCR of inner ear cDNA to detect reduced expression levels of *Grxcr1* in the homozygotes. The Tasmanian devil mutants are available from the European Mouse Mutant Archive (EMMA).

### Preparation of DNA samples and PCR analysis

Genomic DNA was extracted from ear biopsies or post-mortem tail skin using phenol/chloroform extraction with standard protocols. Complementary DNA (cDNA) was prepared after RNA extraction and reverse transcription. Whole inner ears from littermate heterozygotes and *tde/tde* mice and C57BL/6N wild-type mice were dissected and stored in RNA later stabilization reagent (QIAgen) at -20°C. Total RNA was extracted using QIAshredder columns and RNeasy mini kit (QIAgen) according to manufacturer’s instructions. DNAse I (Invitrogen) treatment was used to remove DNA. Reverse transcription reactions were performed using Superscript II First-strand synthesis system kit (Invitrogen).

### Design of primers for mouse Grxcr1 gene

Primers for the genomic or the transcript sequence of the mouse *Grxcr1* gene (ENSMUSG00000068082; Ensembl version 56) were designed using Primer3 software [23]. Transcript primers are located in exons 1 and 4, and are 134kb apart in the genome, ensuring that only cDNA could be amplified from them. The sequences of the primers are detailed in Table 1. PCR was performed using a touchdown protocol:

1. 94°C for 2 minutes
2. 94°C for 30 seconds
3. 64°C for 45 seconds (decreasing by 0.5C per cycle)
4. 72°C for 45 seconds
5. Repeat 2–4 15 times
6. 94°C for 30 seconds
7. 55°C for 45 seconds
8. 72°C for 45 seconds
9. Repeat 6–8 20 times)
10. 72°C for 7 minutes

**Table 1:** List of primers for amplification of Grxcr1 by PCR.

Name	Forward primer	Reverse primer	Product size
**Grxcr1exon1**	5’-TTGATAGCCAAGGCACACTG-3’	5’-AGGAGAAGAGCGCAGAACAG-3’	**589bp**
**Grxcr1exon2**	5’-CACCTCAATGTTTTCCTTCCA-3’	5’-ACGCAAAGGCAGTTTTCTTC-3’	**299bp**
**Grxcr1exon3**	5’-TCTTTTCTCCCTAACCCTCTTG-3’	5’-GGCAGGTGGTACCCAATTAC-3’	**243bp**
**Grxcr1exon4**	5’-TTCTGTTTCTTGTCACCCTATCA-3’	5’-CAACAAAAATTGATTGTAGCCAAA-3’	**372bp**
**Grxcr1—transcript**	5’-GCTGGTATTAGCCAGGACAGCCAGC-3’ (exon 1)	5’-GGAAGCCAAAGCCTCCACAGGAAGG-3’ (exon 4)	**503bp**
**Grxcr1–5’RACE**		5’-GGAAGCCAAAGCCTCCACAGGAAGG-3’ (exon 4)	
		5’-CTTCCGTGGCACACAGAGCATGG-3’ (exon 4)	
		5’-GAGGCTTTCTCTAGGCAGAAGCTCC-3’ (3’UTR-exon 4)	
**Grxcr1—*In situ* hybridization**	5’-GCTGGTATTAGCCAGGACAGCCAGC-3’ (exon 1)	5’-CTTCCGTGGCACACAGAGCATGG-3’ (exon 4)	**568bp**

### Analysis of the sequences

PCR products were separated in agarose gels and purified (QIAgen) according to the manufacturer’s protocol. The purified samples were used for capillary sequencing and the results analysed using DNAStar Lasergene 8 software.

### Scanning electron microscopy (SEM)

Mice at postnatal day 7 (P7; +/+ and +/*tde* n = 15; *tde/tde* n = 12 mice) or at P4 (+/+ and +/*tde* n = 9; *tde/tde* n = 3 mice) were selected for SEM. Inner ears were dissected and fixed in 2.5% glutaraldehyde in 0.1M phosphate buffer for 3 hours at room temperature, followed by washes in 0.1M phosphate buffer. Samples were then prepared using the osmium tetroxide-thiocarbohydrazide method (OTOTO) [24]. Processed cochleae were dehydrated followed by critical point drying using pressurized carbon dioxide. Samples were mounted and examined under a scanning electron microscope (Hitachi 4800 FE) at 5 kV. Images were processed using Adobe Photoshop, and all adjustments to settings were applied equally to control and mutant images.

### Quantitative real-time PCR

Inner ears were dissected and RNA was extracted from whole inner ears at P7 (+/*tde*: n = 5; *tde/tde*: n = 11 mice) and at P12 (n = 2 *+/tde*; n = 4 *tde*/*tde* mice). Total RNA was isolated using RNeasy Mini Kit (Qiagen, Valencia, CA, USA) following the manufacturer’s instructions. RNA was first normalised, then treated with DNase I (Sigma, UK) to remove any remaining DNA, followed by reverse-transcription to cDNA using Superscript II-reverse transcriptase (Invitrogen, Carlsbad, CA, USA) and a poly(dT) primer (Invitrogen, cat no 18418012). Quantitative Real Time PCR (qRT-PCR) was carried out on an ABI Prism 7000 Sequence Detection System (Applied Biosystems) using TaqMan reagents and probes (*Grxcr1* (Applied Biosystems: Mm00473947_m1), *Hprt*, (Mm01318747_g1), *Kcnq4* (Mm01185500_m1) and *Prestin* (552740-C3)). Relative expression levels of each transcript were calculated using the 2^-ΔΔCt^ equation [25] and normalized to *Hprt* (a house-keeping gene). Each sample was run with 3 technical replicates and compared to littermates. The Wilcoxon rank sum test (Mann-Whitney U test) was chosen to determine significance, because it is a suitable test for small sample sizes and populations of unknown characteristics [26].

### 5’ RACE

5’ RACE PCR (5’ rapid amplification of 5’ cDNA ends) was performed to analyse the mRNA structure and expression of *Grxcr1* by generating full-length cDNA from the mouse *Grxcr1* gene. This technique involves three sequential enzymatic steps: reverse transcription from a primer designed to match the 3’ end of the transcript (3’UTR-exon 4, Table 1) followed by degradation of the mRNA template; addition of homopolymeric poly-A tail to the cDNA (5’ end of the transcript); and PCR using a poly(dT) primer to match the poly-A tail and a specific primer nested inside the first primer (Table 1). The resulting 5’ RACE products were cloned into an appropriate vector for sequencing and subsequent manipulation. We used a 5’/3’ RACE Kit, 2^nd^ generation (Roche) and specific primers (Table 1) including one located in the 3’UTR (exon 4) region of the mouse *Grxcr1* transcript and the mRNA from wild-type C57BL/6N mice at P0 and P6 (n = 2 mice), and a heterozygous adult.

### Immunofluorescence

Inner ears from P5-P12 mice were fixed in 4% paraformaldehyde for 2 hours at room temperature. After dissection in PBS, the organ of Corti was washed and permeabilized in 1% PBS/Triton-X-100 (PBT) before blocking with 10% sheep serum. The samples were incubated overnight at 4°C with the primary antibodies: EPS8 (Abcam, ab96144); MYO7A (Proteus Bioscience, 25–6790); ESPN (gift from Bechara Kachar and described in ref [27]); or PTPRQ (gift from Guy Richardson and described in ref [21]). Samples were then washed with PBT and incubated with anti-rabbit Alexa Fluor 488 secondary antibody (Invitrogen, anti-rabbit, diluted 1:300) and rhodamine/phalloidin (Invitrogen, diluted 1:200). Samples were mounted in Prolong Gold mounting medium (Molecular Probes). Images were acquired on either an LSM 510 Meta confocal microscope (Zeiss, Welwyn Garden City) or a Nikon A1R Confocal microscope, and post-acquisition image analyses were performed using Adobe Photoshop CS6.

### Immunohistochemistry

Inner ears from P12 mice were fixed in 4% paraformaldehyde in PBS overnight at 4°C. Samples were embedded in paraffin wax, cut at 8 μm in a mid-modiolar plane and immunolabelled using the Ventana Discovery system (Ventana Medical Systems, Inc. Illkirch, France) according to manufacturer’s instructions. Antibodies to Kcnq4 (Santa Cruz, polyclonal goat, sc-9385) and prestin (Santa Cruz, polyclonal goat, sc-22694) were used as primary antibodies followed by anti-goat secondary antibody (Jackson ImmunoResearch, 705-065-147). For prestin, +/*tde* n = 2; *tde/tde* n = 5 mice. For Kcnq4, +/*tde* n = 2; *tde/tde* n = 2 mice.

### Single-hair cell electrophysiology

Acutely dissected organs of Corti were used to study inner and outer hair cells (IHCs: *n* = 37; OHCs, *n* = 54) from Tasmanian devil mice and control littermates from postnatal day 7 (P7) to P13 for OHCs and P5 to P21 for IHCs, where the day of birth is P0. Animals were killed by cervical dislocation. Cochleae were dissected in normal extracellular solution (in mM): 135 NaCl, 5.8 KCl, 1.3 CaCl_2_, 0.9 MgCl_2_, 0.7 NaH_2_PO_4_, 5.6 D-glucose, 10 Hepes-NaOH. Sodium pyruvate (2 mM), MEM amino acids solution (50X, without L-Glutamine) and MEM vitamins solution (100X) were added from concentrates (Fisher Scientific, UK). The pH was adjusted to 7.5 (osmolality ~308 mmol kg^-1^). Recordings were performed either at room temperature (22-24°C) or at body temperature (34-37°C).

Voltage and current clamp recordings were performed using the following intracellular solution in the patch pipette containing (in mM): 131 KCl, 3 MgCl_2_, 1 EGTA-KOH, 5 Na_2_ATP, 5 Hepes-KOH, 10 Na_2_-phosphocreatine (pH 7.3; osmolality ~296 mmol kg^-1^). Exocytosis and mechanoelectrical transduction recordings were measured using the following intracellular solution (in mM): 106 Cs-glutamate, 20 CsCl, 3 MgCl_2_, 1 EGTA-CsOH, 5 Na_2_ATP, 0.3 Na_2_GTP, 5 Hepes-CsOH, 10 Na_2_-phosphocreatine (pH 7.3). Patch pipettes were coated with surf wax (Mr Zoggs SexWax, USA) to minimise the fast patch pipette capacitance transient. An Optopatch (Cairn Research Ltd, UK) amplifier was used to perform electrophysiological recordings. Data acquisition was controlled by pClamp software using Digidata 1440A boards (Molecular Devices, USA).

The recorded basolateral membrane currents were low-pass filtered at 2.5 kHz (8-pole Bessel), sampled at 5 kHz and stored on computer for off-line analysis (Origin: OriginLab, USA). Membrane potentials in voltage clamp were corrected for the voltage drop across the uncompensated residual series resistance (*R*_s_: 1.5 ± 0.2 MΩ, *n* = 63, after up to 80% compensation) and for a liquid junction potential (K^+^- and Cs^+^-based intracellular solution: –4 mV and –11 mV, respectively).

Real-time changes in membrane capacitance (Δ*C*_m_) were measured using a hardware method (Optopatch). A 4 kHz sine wave of 13 mV RMS was applied to IHCs from −81 mV and was interrupted for the duration of the voltage step (100 ms and in 10 mV increments). The capacitance signal from the Optopatch was amplified (×50), filtered at 250 Hz, and sampled at 5 kHz. Δ*C*_m_ was measured by averaging the *C*_m_ trace over a 200 ms period following the voltage step. Δ*C*_m_ was recorded while applying 30 mM TEA and 15 mM 4AP (Fluka, UK) and additionally 60 μM linopirdine and 300 nM apamin (Tocris, UK) to reduce K^+^ currents. Synaptic transfer relations were fitted using a power function Δ*C*_m_∝*I*_Ca_^N^ (Eq 1), where *N* is the power. The average *N* values reported are from fits to all individual cells tested.

Mechanoelectrical transducer (MET) currents were elicited by stimulating the hair bundles of OHCs using a fluid jet from a pipette (tip diameter 8–10 μm) driven by a piezoelectric disc). The pipette tip of the fluid jet was positioned near to the bundles to elicit a maximal MET current. Mechanical stimuli were applied as force-steps or saturating 50 Hz sinusoids (filtered at 0.25 kHz, 8-pole Bessel) with driving voltages of ± 40 V. Membrane potentials were corrected for the liquid junction potential (Cs-based intracellular solution: –11 mV). Voltage clamp protocols are referred to a holding potential of –81 mV. The peak MET current-voltage curves were fitted according to a simple single-energy-barrier model [28]: *I*(*V*) = *k* [exp ((1 − γ)(*V* − *V*_r_) /*V*_s_) − exp (− γ(*V* − *V*_r_) /*V*_s_)] (eqn.2), where *k* is a proportionality constant, *V*_r_ is the reversal potential, *V*_s_ is a measure for the steepness of the rectification, and γ is the fractional distance within the membrane’s electrical field of an energy barrier, as measured from the outside.

Statistical comparisons of means were made by Student’s two-tailed *t*-test. Mean values are quoted ± s.e.m. where *P* < 0.05 indicates statistical significance.

## Results

### Tasmanian devil mutants are deaf and have abnormally thin stereocilia

**Fig 1:**
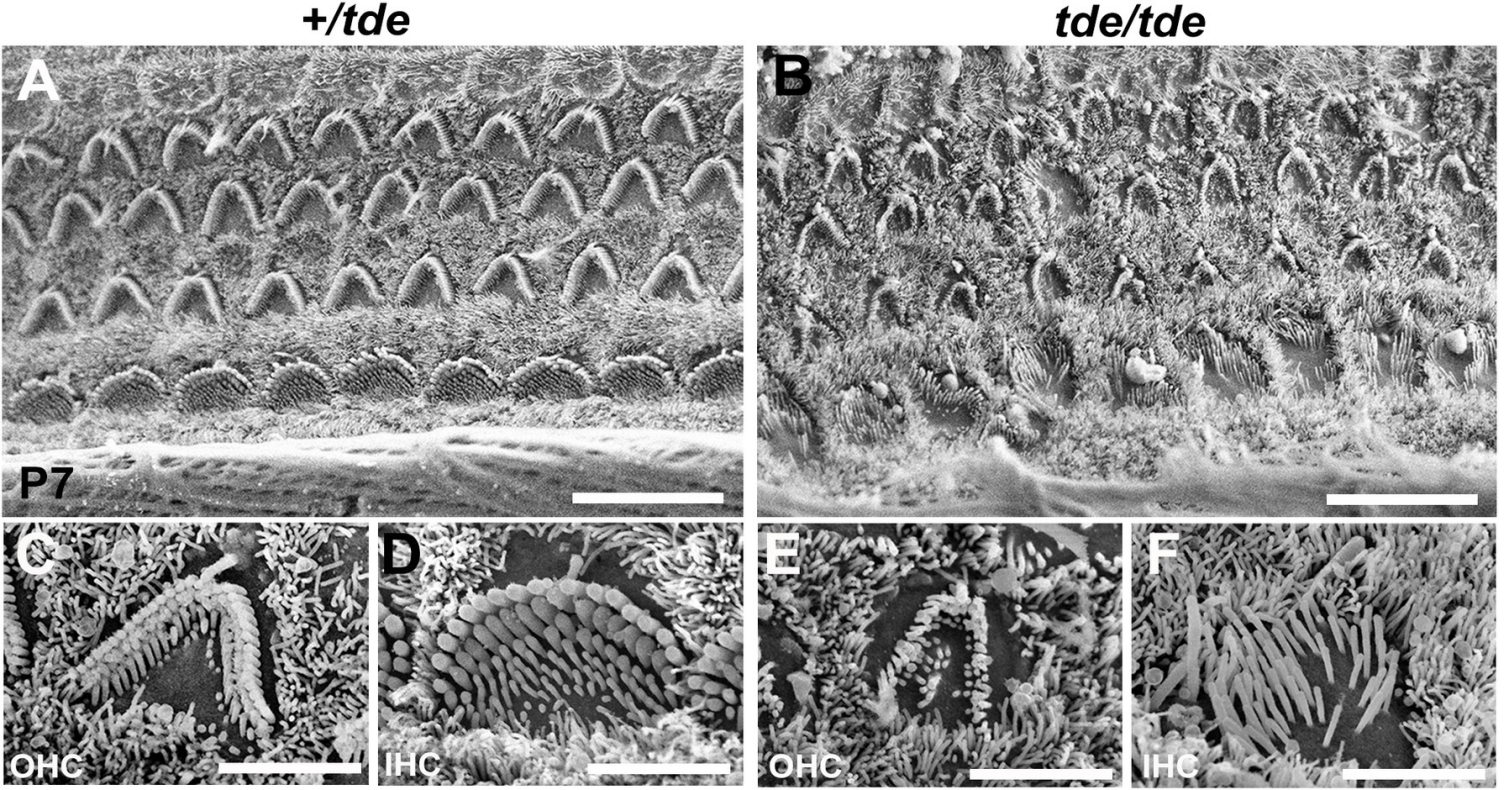
Analysis of cochlear hair cells and their stereocilia. **A-F,** SEM images of the organ of Corti at 50% distance from the base of the cochlea at P7. The typical arrangement of three rows of outer hair cells (OHC) and one row of inner hair cells (IHC) is observed in +/*tde* (**A, C, D**) and *tde/tde* mice (**B, E, F**). The stereocilia in mutants (**B, E, F**) look severely disorganized, abnormally thin with floppy appearance compared to controls (**A, C, D**). Scale bars: (**A, B**) 10 μm; (**C, D, E, F**) 3 μm.

As described previously [10, 14, 15], Tasmanian devil (*tde*) homozygous mutants display hyperactivity, head-bobbing and circling, indicative of vestibular defects, and they lack a Preyer reflex (ear flick in response to noise) and cochlear responses to sound suggesting a severe hearing impairment. Scanning electron microscopy of the organ of Corti at P7 confirmed that the stereocilia of *tde* homozygous (*tde*/*tde*) mice were abnormally thin with a flaccid and disorganized appearance in both inner hair cells (IHC) and outer hair cells (OHC) compared to control heterozygous (+/*tde*) mice (Fig 1).

### Reduced expression of *Grxcr1* mRNA in the inner ear of Tasmanian devil mice

**Fig 2:**
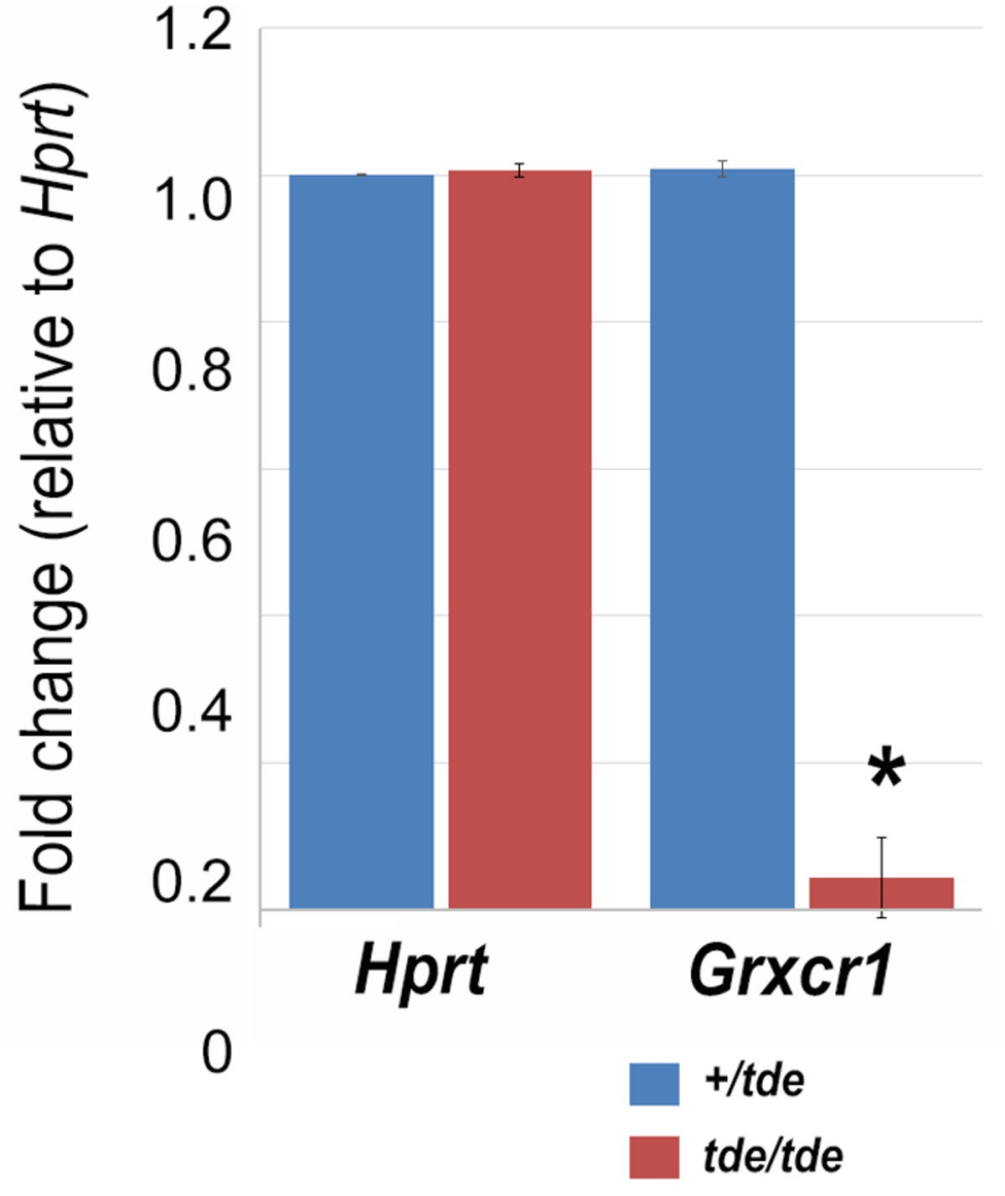
*Grxcr1* expression analysis by *qRT-PCR* in the inner ear. Quantitative real-time PCR on cDNA generated from total RNA from whole inner ear at P7 (n = 11 *tde/tde*; n = 5 +/*tde*). *Grxcr1* normalized to *Hprt* levels was significantly down-regulated in *tde/tde* mice (red bars) compared to controls (blue bars) (*P<0.001, Wilcoxon rank sum test). Error bars indicate standard deviations.

GRXCR1 has previously been shown to be specifically expressed in the cochlea and localized to the neuroepithelium containing hair cells and supporting cells of pre-hearing mice at P5 [10]. We used the gEAR (http://umgear.org) to look at *Grxcr1* expression in single cell and bulk RNAseq datasets at P0 [29, 30] and P1 [31] and found it was strongly expressed in hair cells at these stages. We carried out a quantitative analysis of *Grxcr1* mRNA levels in the inner ear of *tde/tde* and control littermates at P7. We found that the expression of *Grxcr1* was almost completely abolished in *tde/tde* inner ears at P7 (Fig 2; P<0.001, Wilcoxon rank sum test, +/*tde*: n = 5; *tde/tde*: n = 11).

### Molecular analysis of the *Grxcr1* gene in Tasmanian devil mice

**Fig 3:**
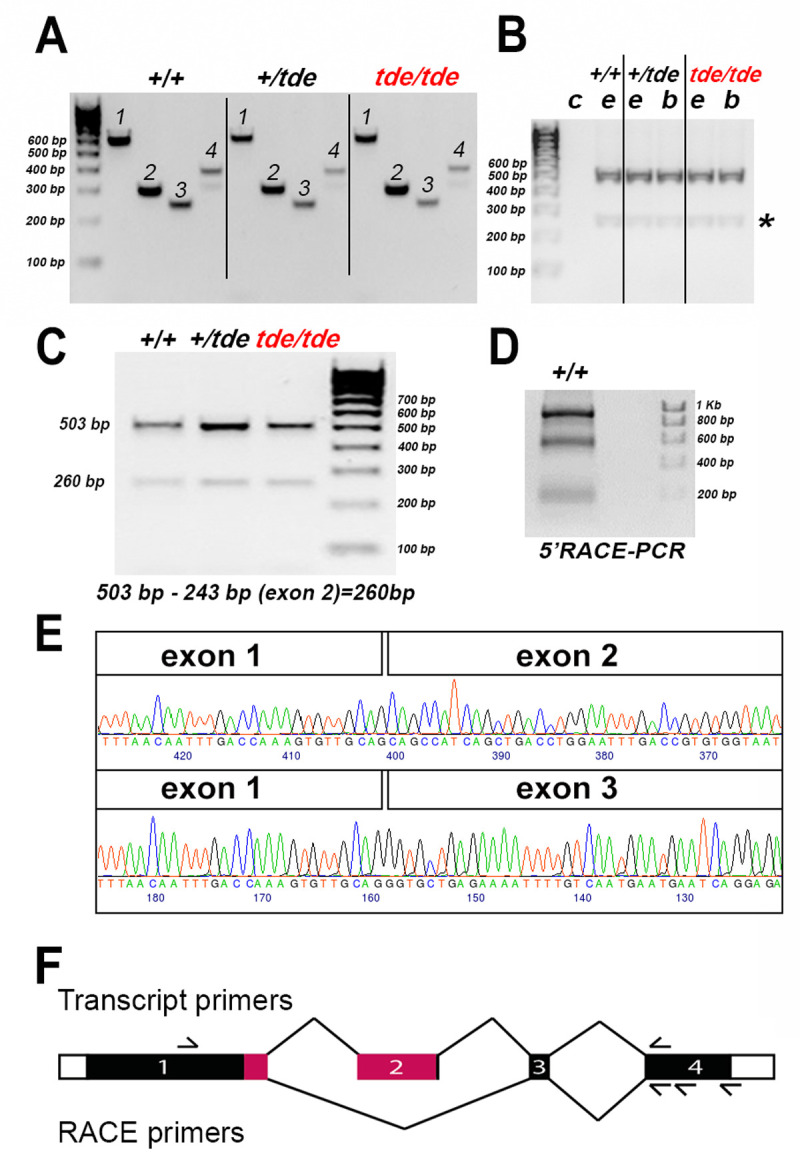
*Grxcr1* gene and transcript expression by PCR. **A,** PCR amplification of genomic DNA from +/+, +/*tde*, *tde*/*tde* mice with primers specific to each exon of *Grxcr1* gene. One single band was amplified for each exon (1–4). Sequencing of all *tde* genotypes revealed no differences between the genotypes. **B**, PCR amplification of cDNA derived from the inner ear (e) and brain (b) tissue of +/+, +/*tde* and *tde*/*tde* adult mice, using primers located in *Grxcr1* exons 1 and 4, resulted in two bands. No band was detected in the negative control (c). The second weak band was detected in all *tde* genotypes (asterisk). **C**, PCR was performed using cDNA from adult inner ears of all *tde* genotypes. Two bands were amplified in mutants and control mice. **D** 5’ RACE-PCR was performed using primers within exon 4 to amplify the entire *Grxcr1* transcript from the P6 C57BL/6N wild-type inner ear. The gel shows 2 strong bands of different sizes that were sequenced. **E**, Partial traces and sequence of the larger band (upper panel) and smaller band (lower panel) amplified in B and C showing part of the *Grxcr1* transcript sequence which skips exon 2. **F**, Schematic of the *Grxcr1* gene, not to scale. Protein-coding sequences are shown as filled rectangles, and untranslated sequences as empty rectangles. The exons are numbered, and lines connect them in the two splicing patterns observed, with the canonical pattern on top and the novel pattern below. The approximate positions of the transcript primers are shown above, and the RACE primers below. Pink indicates the location of the glutaredoxin domain (amino acids 127–234, from Uniprot, www.uniprot.org, accessed July 2021).

Tasmanian devil mice arose when a RSV-PALP transgene became inserted by accident into the first intron of *Grxcr1* [10, 14, 22]. To analyse the effect of this transgene insertion on the genomic and transcript sequence of *Grxcr1* in *tde* mutant mice, we performed PCR analysis and subsequent sequencing of the amplified bands (see **Table 1** for the primers). One transcript of *Grxcr1* is described in Ensembl: Grxcr1-201 (ENSMUST00000094715); 1,071 base pairs in 4 exons), which is translated into a protein of 296 amino acids (www.ensembl.org, accessed June 2020). We analysed the genomic sequence of all four coding exons of the *Grxcr1* gene using primers specific for each exon (**Table 1**), covering the entire coding region and the splice sites, using genomic DNA from phenotyped adults (n = 1 +/+ (C57BL/6N); n = 12 *+/tde*; n = 16 *tde*/*tde* mice). Exons were sequenced by capillary sequencing and traces were analysed. No changes were found in the coding sequence of any exon of the *Grxcr1* gene in the homozygous mutants compared to wild-type C57BL/6N and *+/tde* mice (Fig 3A). PCR amplification of total cDNA from adult inner ears and brains using primers located within *Grxcr1* exons 1 and 4 showed that two bands of different size were amplified in both mutant and control samples (Fig 3B and 3C). To find out whether the two amplified bands corresponded to two different transcripts, we performed 5’ RACE-PCR (PCR for rapid amplification of cDNA ends) from inner ear samples of C57BL/6N wild-type mice at P0 and P6, using a primer in the 3’UTR for the initial reverse transcription. The sequencing of the two amplified bands revealed that the larger band contained all four exons whereas the smaller band skipped exon 2 and is therefore a novel transcript (Fig 3D and 3E; S1 File). This smaller, novel transcript, which we observed in cDNA made using a poly-d(T) primer as well as in the 5’RACE, had clearly undergone intron excision, so we conclude that it is likely to represent the mature mRNA sequence. Therefore, our results show the presence of two different transcripts of *Grxcr1*, which are identical in control and *tde/tde* inner ears. Exon 2 has 243 base pairs and starts and ends in phase 0, so its absence does not change the amino acid sequences of exons 1, 3 or 4, but the resulting protein would lack most of the glutaredoxin domain so likely has a different function to the full length GRXCR1 (Fig 3F; S1 File). In conclusion, while the transgene insertion causes a considerable reduction of *Grxcr1* expression, it does not affect the transcript sequence.

### Key hair bundle proteins are distributed normally in Tasmanian devil stereocilia

The main phenotype characterizing *tde/tde* is the presence of abnormally thin stereocilia with a flaccid appearance in cochlear and vestibular hair cells (Fig 1B, 1E and 1F). The stereocilia bundle shows progressive disorganisation from birth onwards, ultimately leading to extensive hair cell degeneration in adults [14]. Therefore, we analysed the expression patterns of several key proteins known to play a role in the organization of the stereociliary actin core in the development of the hair bundle: ESPN, MYO7A, EPS8 and PTPRQ. We used immunostaining and confocal microscopy on whole-mount preparations of the organ of Corti from P5-P12 mice to analyse the distribution of these proteins. The disorganisation of the stereocilia makes assessment of labelling challenging, so we have focussed on comparing the general features of labelling between mutants and controls.

**Fig 4:**
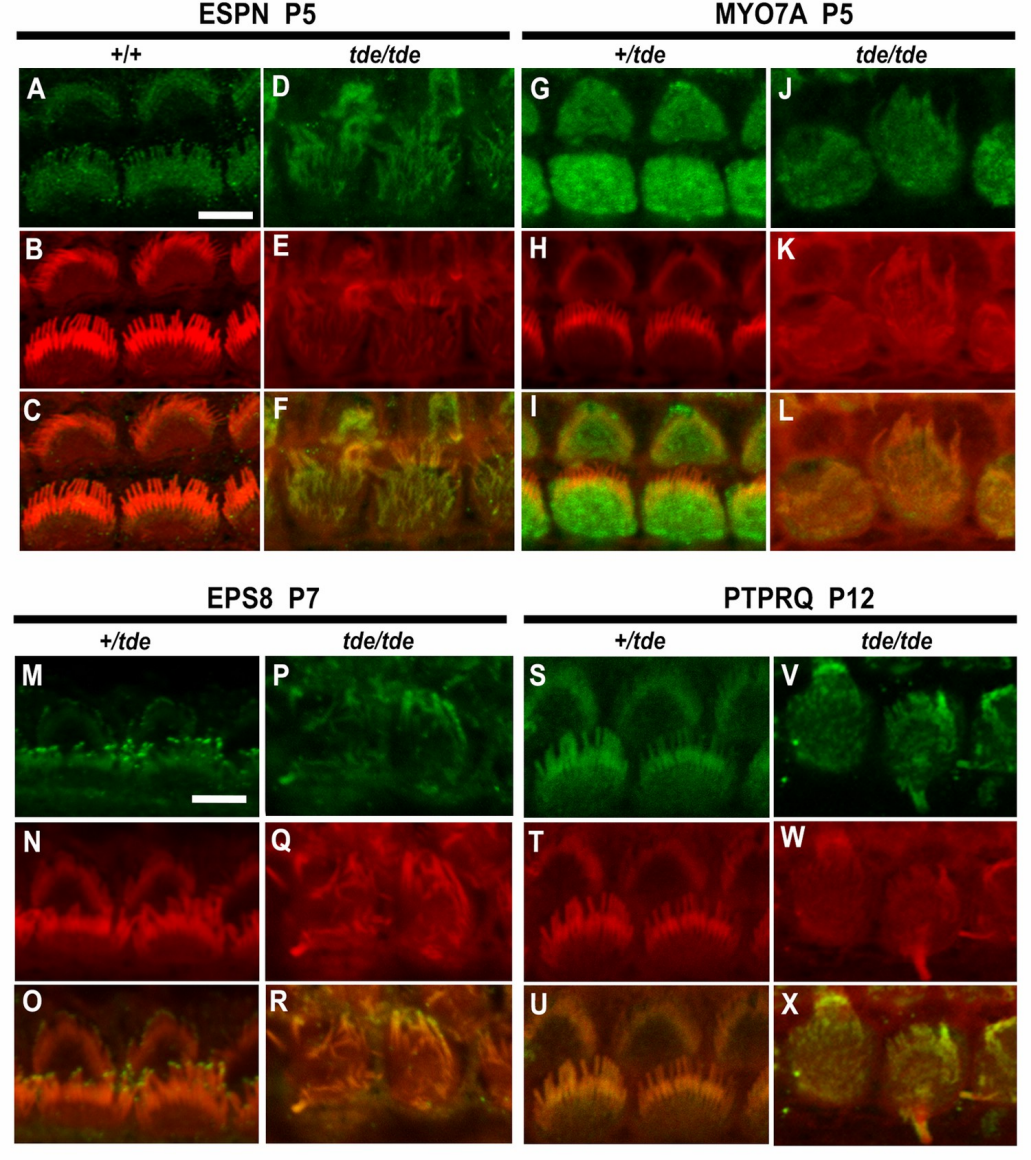
Analysis of hair bundle protein expression by immunofluorescence. Inner ear tissue from +/*tde* and *tde*/*tde* at P5-P12 were incubated with antibodies for hair bundle proteins ESPN, MYO7A, EPS8 and PTPRQ (in green), rhodamine-phalloidin (in red) and merged images (yellow) obtained by confocal microscopy. Images show inner hair cell (IHC) staining in the middle turn of the cochlear duct. **A-F**, ESPN is expressed in a punctate manner along the length of stereocilia in controls and *tde* mutants (P5, +/+ and +/*tde* n = 3, *tde/tde* n = 6 mice). **G-L**, MYO7A is expressed widely within the top of the hair cell of *tde* mutants in a broadly similar way to heterozygotes (P5, +/*tde* n = 4, *tde/tde* n = 3 mice). **M-R**, EPS8 immunoreactivity is detected at the tips of stereocilia in heterozygous mice and also in the tips of the thinner and more disorganized stereocilia of *tde* mutants (P7, +/*tde* n = 5, *tde/tde* n = 4 mice). **S-X**, PTPRQ expression is found throughout the length of stereocilia in heterozygous mice and a broadly similar distribution is also observed in *tde* mutants (P12, +/*tde* n = 4, *tde/tde* n = 1 mice). Scale bars: 5 μm.

ESPN is an actin bundling protein expressed in the core of stereocilia and plays an important role in hair bundle development [32, 33]. Our results indicate that ESPN is expressed in a punctate way along the entire length of the stereocilia (Fig 4A–4F) of *tde/tde* and control hair cells. The punctate labelling has recently been described elsewhere [34].

MYO7A is an unconventional myosin expressed in sensory hair cells and stereocilia, and known to be involved in developing the correct hair bundle architecture [19, 35]. Mice deficient in MYO7A (*shaker1* mutants) show severe hair bundle disruption and abnormally elongated stereocilia [19, 36], which are also characteristics present in hair cells from *tde/tde* mice. The pattern of expression of MYO7A in the hair cells was broadly similar in *tde/tde* and control mice, with labelling evenly distributed within the top of the hair cell (Fig 4G–4L**).**

EPS8 is an evolutionarily conserved signal transducer known to have multiple functions in the control of actin dynamics. *Eps8* mutant mice have shorter and more numerous stereocilia than those of control mice [37]. The localization of labelling was observed at the tips of stereocilia (Fig 4M–4R) in both *tde/tde* mutants and littermate controls.

PTPRQ is a protein generally concentrated at the base of stereocilia and is required for their normal structure and function [38, 39]. Mutations in *Ptprq* lead to different degrees of hair bundle disorganization and formation of giant stereocilia, and hearing loss [21, 39], as seen in *tde/tde* mice [14]. We found that the distribution of PTPRQ in the stereocilia of *tde/tde* mice was generally similar to that of littermate controls (Fig 4S–4X).

### Mechanoelectrical transducer current is reduced in Tasmanian devil homozygotes

**Fig 5:**
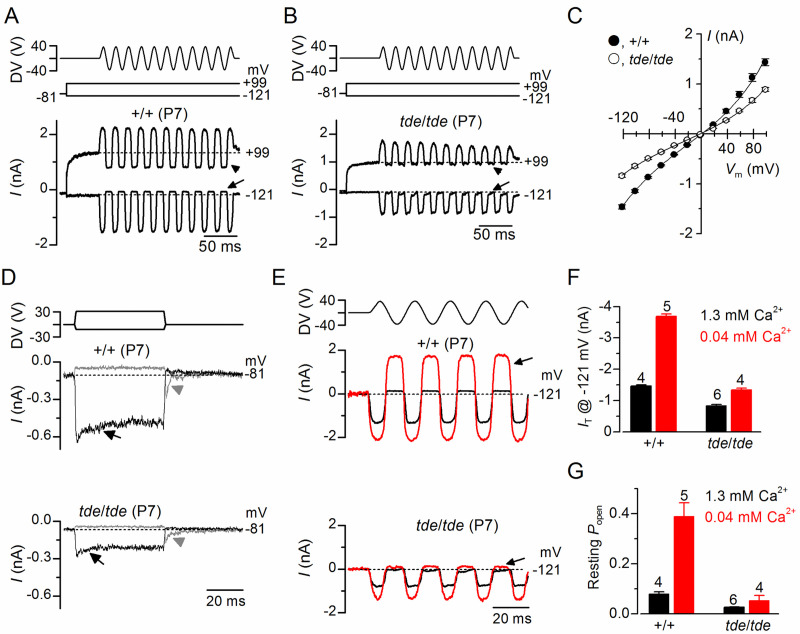
Mechanoelectrical transducer currents in Tasmanian devil inner hair cells. **A** and **B**, Saturating transducer current recordings from an apical-coil control (***A***; +/+) and a mutant (**B**; *tde*/*tde*) P7 OHC in response to a sinusoidal force stimulus of 50 Hz to the hair bundles. The fluid jet driver voltage (DV) of ± 40 V is shown in the top panels (negative deflections of the DV are inhibitory). OHC membrane potential was held at −81 mV and depolarized in 20 mV increments from −121 mV (for clarity only responses to −121 mV and +99 mV are shown). The arrows and arrowheads indicate the closure of the transducer channels, i.e. disappearance of the resting current, during inhibitory bundle displacements. Dashed lines indicate the current at the different test potentials. Note that there is no or very little resting transducer current in the *tde*/*tde* OHC at either potential. In controls the resting current increases with membrane depolarization. **C**, Peak-to-peak transducer current-voltage curves were obtained from 4 control and 5 mutant OHCs (P7) in 1.3 mM extracellular Ca^2+^. The data were fit according Eq 2 (see Methods) with values: control *k* = 405 ± 25, *V*_r_ = 0.1 ± 0.5 mV, *V*_s_ = 40 ± 2 mV, and γ = 0.43 ± 0.01; mutant *k* = 266 ± 23, *V*_r_ = -2.1 ± 1.0 mV, *V*_s_ = 43 ± 3 mV, and γ = 0.43 ± 0.01. **D,** Step deflections of the OHC hair bundle (fluid jet driver voltages are shown in the top panel) elicited transducer currents recorded at –81 mV from a control (+/+) and a mutant (*tde*/*tde*) OHC. Excitatory bundle deflection (positive DV) elicited inward transducer currents that declined or adapted over time in control and mutant OHCs (black arrows). Inhibitory bundle deflection (negative driver voltage and grey current traces) turned off the resting transducer current (indicated by the dashed lines) in control cells but this was almost negligible in *tde*/*tde* cells. The transducer current in both control and mutant OHCs showed evidence of rebound adaptation (grey arrowheads) upon termination of the inhibitory stimulus. **E**, Saturating transducer currents recorded at a holding potential of −121 mV (fluid jet driver voltage above) from a control (+/+) and a mutant (*tde*/*tde*) OHC in the presence of 1.3 mM (black traces) and 0.04 mM (endolymph-like; red traces) extracellular Ca^2+^. For comparison the baseline current has been zeroed in both cells so the resting transducer current is now the difference between zero (dashed line) and the positive current during negative bundle stimulation (arrows). **F** and **G**, Maximal transducer current size at the membrane potential (**F**) and resting transducer channel *P*_open_ (**G**) at −121 mV in control and *tde*/*tde* OHCs in the presence of 1.3 mM and 0.04 mM extracellular Ca^2+^. Resting *P*_open_ was calculated as the ratio between resting and maximal transducer currents. Recordings were made at room temperature.

We then investigated whether the abnormal hair bundles of *tde/tde* mice had any physiological consequence by testing their ability to transduce mechanical stimuli. The mechanoelectrical transducer (MET) current was recorded from P7 apical-coil OHCs by displacing their hair bundles using a piezo-driven fluid-jet. Upon moving the bundles in the excitatory direction (i.e. towards the taller stereocilia) and at negative membrane potentials, a large inward MET current could be elicited in OHCs from both control (+/+) and mutant (*tde*/*tde*) mice (Fig 5A and 5B, respectively). However, the maximum MET current at −121 mV was significantly smaller in mutant (−837 ± 34 pA, *n* = 4) compared to control (−1463 ± 41 pA, *n* = 5, *P*<0.0001, *t-test*) mice. Any resting current flowing through open MET channels in the absence of mechanical stimulation was reduced when bundles were moved in the inhibitory direction (i.e. away from the taller stereocilia) in all control and mutant OHCs (Fig 5A and 5B, arrows). The fraction of the resting MET current was also significantly reduced in mutant (0.026 ± 0.002, *n* = 5, at −121 mV) compared to control (0.079 ± 0.008, *n* = 4, *P*<0.0005, *t*-test) mice. Because the MET current reverses near 0 mV, it became outward when excitatory bundle stimulation was applied during voltage steps positive to its reversal potential Fig 5A–5C). At positive potentials, the larger resting transducer current, which was more evident in control OHCs (e.g. +99 mV in Fig 5A and 5B: arrowheads), is due to an increased open probability of the transducer channel resulting from a reduced driving force for Ca^2+^ influx [40, 41].

We then investigated whether the adaptation properties of the MET current were affected in Tasmanian devil mice by stimulating the hair bundles of OHCs using mechanical step stimuli instead of sinusoids. Excitatory bundle movements with non-saturating stimuli elicited rapid inward currents at a holding potential of –81 mV that declined or adapted over time in both control and mutant P7 OHCs (Fig 5D, arrows). Inhibitory hair bundle stimulation shut off the small fraction of the current flowing at rest and at the offset of large inhibitory steps they caused a transient rebound (downward dip: Fig 5D, arrowheads).

We further tested the Ca^2+^ sensitivity of the transducer apparatus by deflecting the bundle in the presence of an endolymph-like solution containing 0.02–0.04 mM Ca^2+^ [42, 43]. Reducing Ca^2+^ influx into the transducer channel by either depolarizing hair cells to near the Ca^2+^ equilibrium potential (as shown in Fig 5A) or lowering the extracellular Ca^2+^ concentration from 1.3 mM to 0.04 mM increased both the resting and the maximal MET current in both control and mutant OHCs (Fig 5E), as previously described in non-mammalian vertebrates and mammals [41, 44, 45]. This is because Ca^2+^ is a permeant blocker of the MET channel and as such the increased MET current amplitude in low Ca^2+^ reflects the partial relief of this block. Moreover, extracellular Ca^2+^, by binding to the MET channel pore [41, 44, 45] or with the lipid environment [46] is able to directly regulate the open probability of the MET channel. Although the size of the MET current was significantly increased in low-Ca^2+^ (*P*<0.0001) in both control and mutant OHCs, the increment was much larger in the former (control: 2.5x; mutant: 1.5x, Fig 5F). The fraction of the resting open MET current was also significantly increased in control OHCs (*P*<0.001) but not in mutant cells (*P*>0.05, one-way ANOVA, Fig 5G). The above results indicate that the size and Ca^2+^ sensitivity of the MET current are affected in the OHCs from *tde/tde* mice.

### IHCs from Tasmanian devil homozygous mice do not develop adult-type ion channels

**Fig 6:**
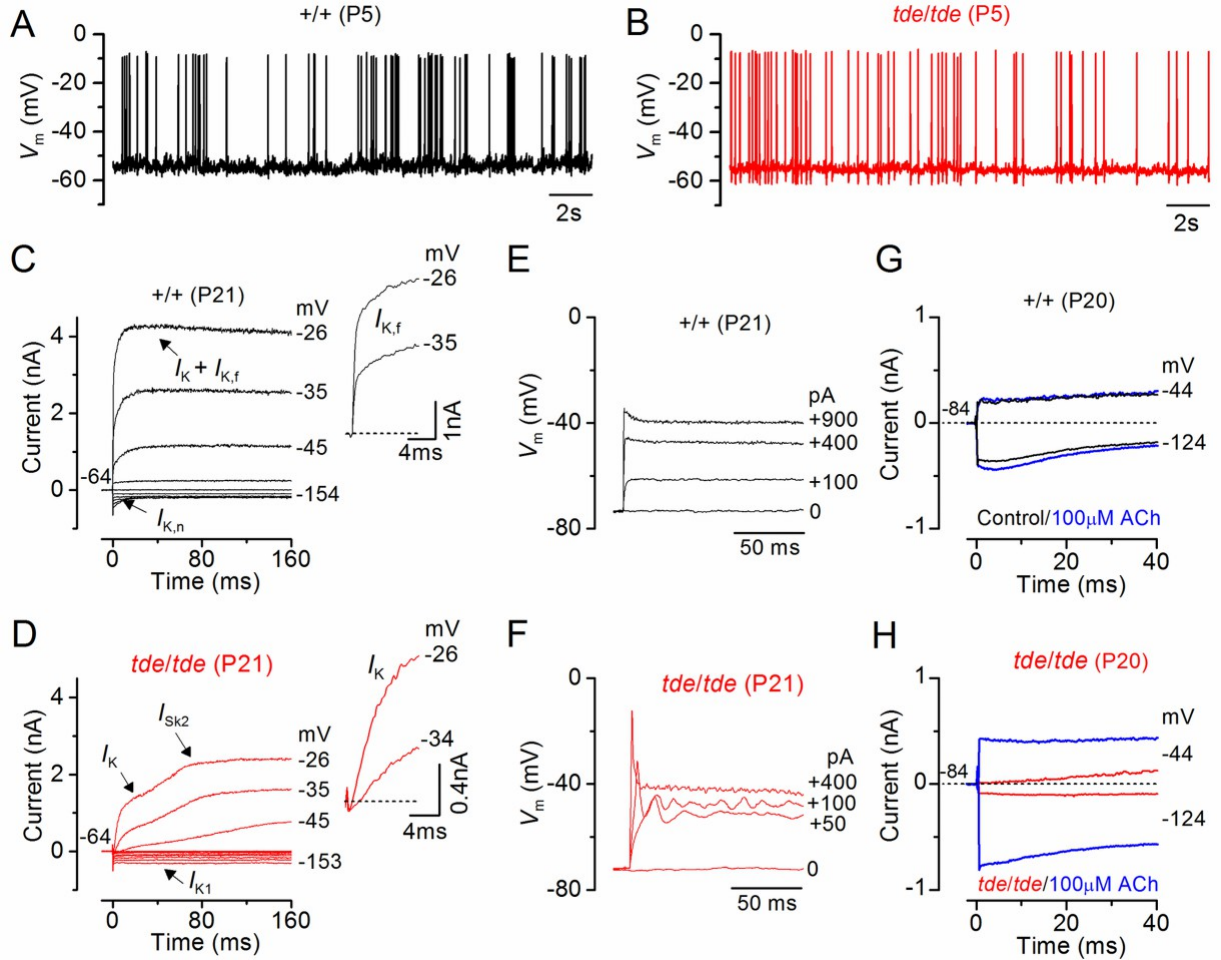
IHC current and voltage responses in Tasmanian devil mice. **A** and **B**, Spontaneous Ca^2+^ dependent action potentials recorded from a control (**A**: black line) and a *tde*/*tde* (**B**: red line) P5 IHC. **C** and **D**, Currents from a control and a *tde*/*tde* adult P21 IHC, respectively, were elicited by depolarizing voltage steps in 10 mV nominal increments from –154 mV to the various test potentials shown by some of the traces; the holding potential was –64 mV. The insets show the same current recordings on an expanded scale (first 25 ms), note that the rapidly activating *I*_K,f_ is only present in control cells whereas an inward current (*I*_Ca_) precedes the activation of the much slower K^+^ current (*I*_K_) in *tde*/*tde* IHCs. **E** and **F**, Voltage responses induced by depolarizing current injections (the level is indicated to the right of the traces) applied to a control and a *tde*/*tde* adult IHC, respectively. Note that the *tde*/*tde* IHC showed a large initial spike followed by membrane potential oscillations compared to the graded responses of the control cell. **G** and **H**, Membrane currents recorded from an adult control and *tde*/*tde* IHC, respectively, in response to a hyperpolarizing and a depolarizing voltage step from the holding potential, before (black/red traces) and during superfusion of 100 μM ACh (blue traces). Current responses were obtained at room temperature while voltage recordings were made at body temperature.

We then investigated whether the biophysical properties of hair cells were affected in Tasmanian devil mice. The resting membrane potentials and size of K^+^ currents recorded from immature (<P12) control (+/+) IHCs were similar to those in *tde*/*tde* cells (**Table 2**). Moreover, all immature IHCs investigated were able to generate spontaneous repetitive Ca^2+^ action potentials (Fig 6A and 6B for control and *tde*/*tde* IHCs, respectively) as previously shown in wild-type cells [47]. These results show that the biophysical properties of pre-hearing IHCs (<P12 in most rodents) are not affected in early postnatal *tde*/*tde* mice. We then investigated whether IHCs were able to acquire functional maturity, which is associated with the down-regulation of immature ion channels and acquisition of adult-like electrical properties including a rapidly activating large conductance Ca^2+^-activated K^+^ current (*I*_K,f_) and a current carried by KCNQ4 channels (*I*_K,n_) with an unusually hyperpolarized activation range. Potassium currents in IHCs were elicited in response to depolarizing voltage steps in 10 mV increments from –154 mV (holding potential –64 mV). Both *I*_K,f_ and *I*_K,n_ were present in adult IHCs (P20-P21) from control (Fig 6C), but absent in cells from *tde*/*tde* mice (Fig 6D; see also Table 2), which instead retained an immature phenotype by expressing the inward rectifier K^+^ current *I*_K1_ and the small conductance Ca^2+^ activated K^+^ current *I*_SK2_. While control IHCs generate fast and graded voltage responses to current injection of up to 900 pA (Fig 6E), as previously described in adult IHCs of normal CD-1 mice [48], mutant cells retained the ability to generate an initial Ca^2+^ action potential (Fig 6F).

**Table 2:** Properties of immature and adult OHCs from Tasmanian Devil mice.

	Immature		Adult			
	IHCs (P5)		IHCs (P20-P21)		OHCs (P12-P13)	
	+/+	*tde*/*tde*	+/+	*tde*/*tde*	+/+	*tde*/*tde*
Membrane capacitance (pF)	7.7 ± 0.2 (3)	6.8 ± 0.2 (5)	8.7 ± 0.6 (11)	7.6 ± 0.3 (10)	8.9 ± 0.2 (13)	8.7 ± 0.2 (14)
Resting potential (mV)	-57.3 ± 0.3 (3)	-56.6 ± 0.7 (5)	-72.7 ± 0.8 (7)	-72.3 ± 4.3 (4)	-72.0 ± 1.4 (4)	-64.8 ± 1.0 (5)
*I*_K1_ at -124 mV (pA)	-210 ± 14 (3)	-170 ± 21 (4)	-	-107 ± 31 (4)	-	?
*I*_K_ at 0 mV (nA)	3.8 ± 0.1 (3)	3.7 ± 0.2 (4)	9.5 ± 1.2 (6)	7.7 ± 0.7 (4)	1.2 ± 0.1 (4)	0.9 ± 0.1 (11)
*I*_K,f_ at -25 mV (nA)	-	-	2.8 ± 0.5 (4)	-	-	-
*I*_K,n_ at -124 mV (pA)	-	-	332 ± 19 (5)	-	185 ± 27 (4)	61 ± 9 (11)
*g*_ACh_ at -84 mV (nS)			-	9.5 ± 2.8 (5)	9.9 ± 1.7 (9)	18.8 ± 1.0 (3)

Values are means ± s.e.m.; number of hair cells is in parentheses. *I*_K1_ = Inward rectifier K^+^ current; *I*_K_ = Delayed rectifier K^+^ current; *I*_K,n_ = Negatively activated K^+^ current carried by KCNQ4 channels; *I*_K,f_ = Ca^2+^-activated K^+^ current. “-“: not present, “?“: not quantified.

Another characteristic of IHC development is that they lose the ability to respond directly to the efferent neurotransmitter acetylcholine (ACh) by P14-P16 [49, 50]. The ACh-activated current in IHCs is mediated by Ca^2+^ entering hair cells through α9α10-nAChRs and activating SK2 channels [51, 52]. In agreement with previous findings, adult control IHCs did not respond to the extracellular application of 100 μM ACh (Fig 6G). In contrast, all mutant IHCs showed a large ACh-activated current at around the holding potential of −84 mV (Fig 6H, see also Table 2), which further supports the immature state of Tasmanian devil mutant IHCs.

We suggest that the immature state of IHCs in *tde* mutants is likely to be the consequence, not the cause, of reduced transduction current by analogy with other recent observations showing that defects in the MET current directly influence IHC development [53].

### Exocytosis in adult IHCs from homozygous Tasmanian devil mice fails to mature

**Fig 7:**
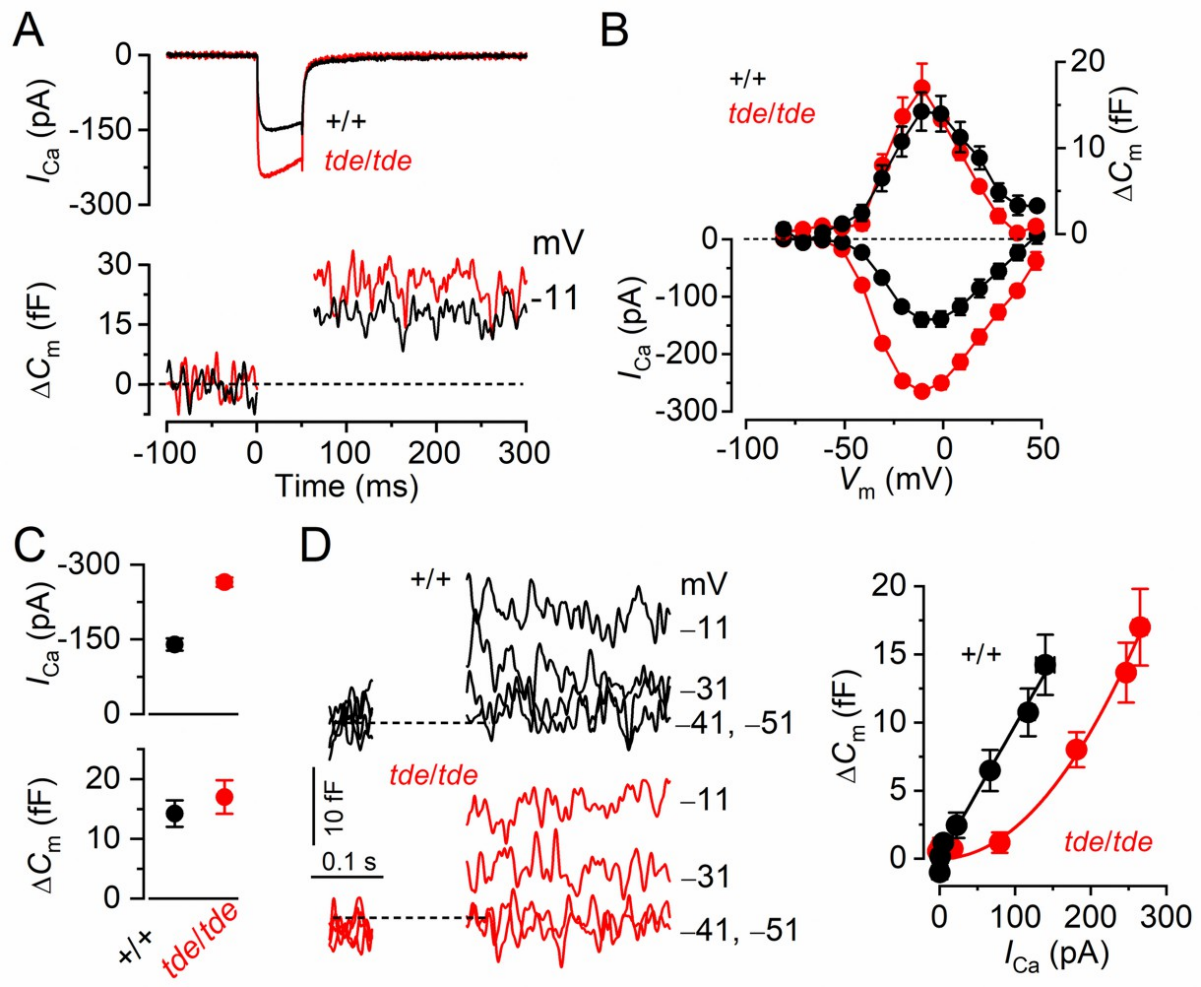
The properties of exocytosis in Tasmanian devil IHCs. **A**, *I*_Ca_ and ΔC_m_ responses from adult control and *tde*/*tde* IHCs. Recordings were obtained in response to 50 ms voltage steps, in 10 mV increments, from −81 mV. For clarity, only maximal responses are shown. **B**, Average peak *I*_Ca_-voltage (left axis) and Δ*C*_m_-voltage (right axis) curves from control (P18, *n* = 4) and *tde*/*tde* (P18, *n* = 4) IHCs. **C**, Maximal peak *I*_Ca_ (top panel) and Δ*C*_m_ (bottom panel) values obtained at −11 mV, from control and *tde*/*tde* IHCs. **D**, Synaptic transfer relations obtained by plotting Δ*C*_m_ against the corresponding *I*_Ca_ between −71 mV and −11 mV (the peak *I*_Ca_) from panel **B**, showing that *tde*/*tde* IHCs exhibited larger *I*_Ca_ values and a steeper Ca^2+^ dependence of exocytosis than control cells. Fits are according to eqn.1 (see Methods). The panels on the left show average Δ*C*_m_ traces from all control and *tde*/*tde* IHCs, the membrane potential is indicated next to the traces. Recordings were made at body temperature.

We investigated whether, in addition to the K^+^ currents, the development of *I*_Ca_ and the induced exocytosis fail to mature in *tde*/*tde* mice. Exocytosis was estimated by measuring increases in cell membrane capacitance (Δ*C*_m_) following depolarizing voltage steps, which is generally interpreted as an indication of neurotransmitter release from presynaptic cells. The synaptic machinery of IHCs becomes more sensitive to Ca^2+^ as they mature, causing synaptic vesicles to be released linearly with increases in Ca^2+^ current. We found that in IHCs from *tde*/*tde* mice the developmental linearization of the exocytotic Ca^2+^ sensitivity did not occur. In adult *tde*/*tde* IHCs the maximal size of the Ca^2+^ current (*I*_Ca_) was significantly larger (*P*<0.0005) than that of control cells (Fig 7A–7C), but similar to that normally seen in pre-hearing cells (e.g. [54]). However, the corresponding Δ*C*_m_ was similar between the two genotypes (Fig 7B and 7C, lower panel). As a consequence the exocytotic Ca^2+^ dependence, defined as the variation in Δ*C*_m_ as a function of *I*_Ca_ and displayed as a synaptic transfer function, was significantly less linear in IHCs from *tde*/*tde* mice (power of 2.02 ± 0.14, *n* = 4) than in control adult IHCs (power of 0.98 ± 0.07, *n* = 4: Fig 7D), and was more similar to that of immature cells [55, 56].

### OHC basolateral membrane properties fail to mature completely in Tasmanian Devil mice

**Fig 8:**
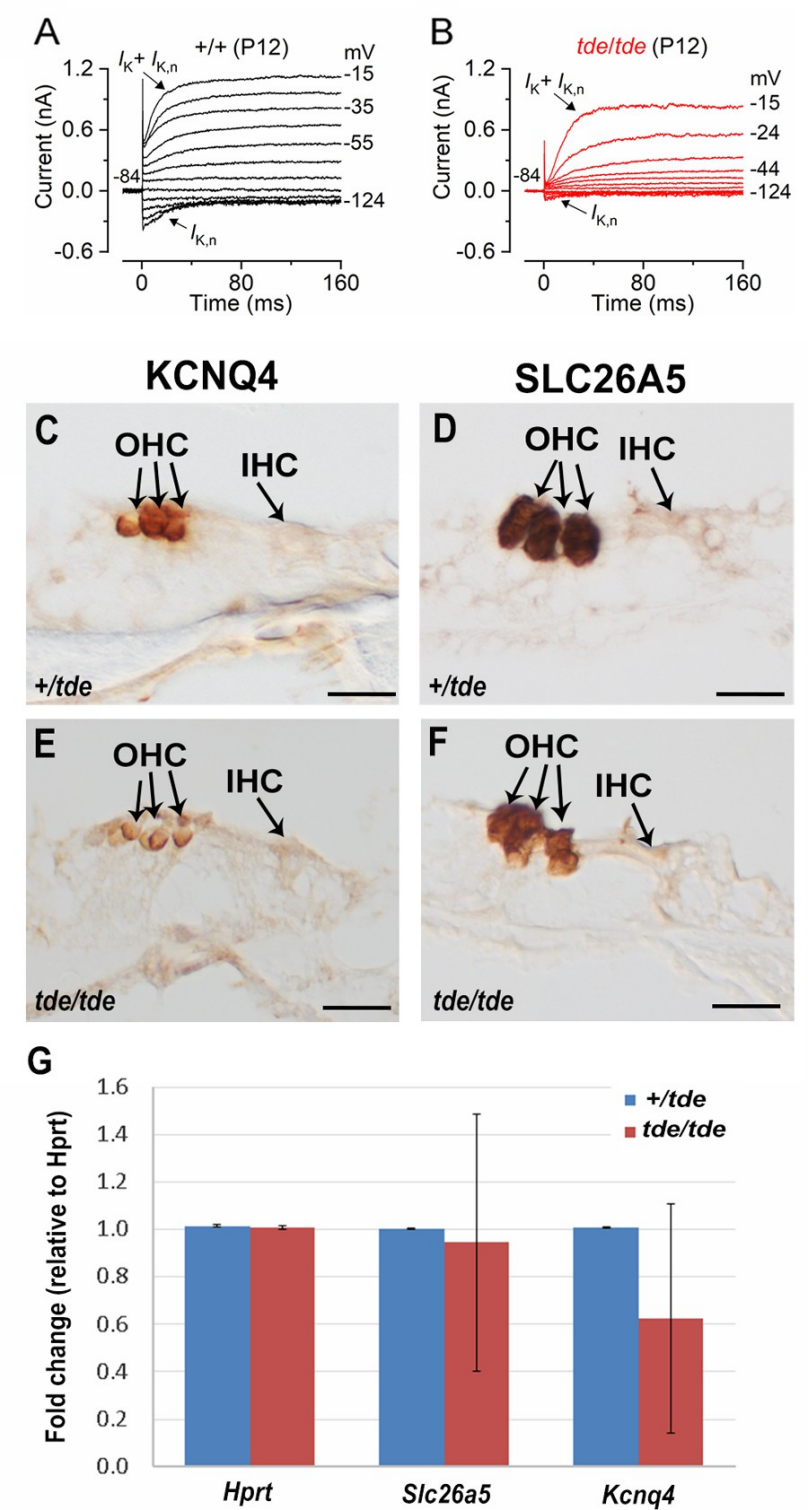
OHC development is affected in Tasmanian devil homozygous mice. **A** and **B**, K^+^ currents recorded from mature control and *tde*/*tde* OHCs, respectively, elicited by depolarizing voltage steps from –124 mV to –15 mV in 10 mV nominal increments from the holding potential of –84 mV. Control cells showed a large negatively activating K^+^ current characteristic of adult OHCs, *I*_K,n_, whereas it was much smaller or almost absent in *tde*/*tde* OHCs. Recordings were made at room temperature. **C-F**, Expression of KCNQ4 and SLC26A5 observed using immunohistochemistry at P12 shows strong labelling in OHCs. **G,** Quantitative real-time PCR on cDNA generated from *Hprt* normalized total RNA from whole inner ear at P12 (n = 4 *tde/tde*; n = 2 +/*tde*). *Kcnq4* and *Slc26a5* are both down-regulated in *tde/tde* mutant mice compared to controls but neither difference is statistically significant (P>0.05, Wilcoxon rank sum test). Technical replicates (3 for each sample) showed minimal variation, but samples were more variable. Error bars represent standard deviations.

We finally investigated whether adult OHCs from Tasmanian Devil mutant mice were also unable to mature as in IHCs. Potassium currents were elicited by applying depolarizing voltage step of 10 mV increments from –124 mV (holding potential –84 mV). The resting membrane potential of mature OHCs was significantly more depolarized in mutant than in control cells (*P*<0.0005, see Table 2). *I*_K,n_, the major current component in adult mouse OHCs, was largely reduced in mutant compared to control cells (Fig 8A and 8B; Table 2). We measured expression levels of two genes that are strongly expressed in outer hair cells, *Slc26a5* (prestin) and *Kcnq4*, and both showed apparently reduced immunolabelling (although this is not a quantitative measure; Fig 8C–8F). qRT-PCR showed that *Kcnq4* in the mutants was 63% of normal levels, although this was not a significant difference (n = 4 *tde/tde*; n = 2 +/*tde*, Wilcoxon rank sum test) (Fig 8G). Any reduced expression of *Kcnq4* would be correlated with the reduced *I*_*K*,*n*_ current recorded from OHCs of *tde/tde* mice (Fig 8B).

## Discussion

We have shown that GRXCR1 is crucial for the growth of the stereociliary bundles in cochlear hair cells, and is required for the functional maturation of these cells into sensory receptors. Despite the thin stereocilia and disorganised hair bundles in the Tasmanian devil (*tde/tde*) homozygous mutant mice, in which *Grxcr1* is severely down-regulated in the inner ear, we found that the expression patterns of four key proteins involved in the elongation and widening of the stereocilia (ESPN, MYO7A, EPS8 and PTPRQ) was generally similar in mutants compared with controls with normal hair bundles. We have demonstrated that mechanoelectrical transducer (MET) currents could still be recorded in pre-hearing OHCs from *tde/tde* mice despite the presence of abnormal stereociliary bundles. However, the MET current was smaller than in littermate controls, with a reduced resting open probability. Both IHCs and OHCs of *tde/tde* mice were unable to become fully functional sensory receptors after the onset of hearing, since they retained the basolateral membrane protein profile of immature cells. We conclude that GRXCR1 plays an essential role in the development of the hair bundle and regulates the morphological and function development of mammalian cochlear hair cells.

### *Grxcr1* is down-regulated in Tasmanian devil mutant mice

In *tde/tde* mice, the chance insertion of the RSV-PALP transgene into intron 1 of the *Grxcr1* gene disrupts its expression. In 5’ RACE PCR experiments, we showed the presence of two different *Grxcr1* transcripts in the inner ear of wild-type mice, including a new transcript that skips exon 2. The sequences of these transcripts were not affected in *tde* mutants. However, quantitative analysis of *Grxcr1* transcripts revealed a considerably reduced level in *tde* mutants. In pirouette (*Grxcr1*^*pir*^) mice the same intron is affected [10], suggesting that this chromosomal region is critical for normal *Grxcr1* expression. Low amounts of mRNA suggest residual GRXCR1 protein might be expressed in hair cells, but not enough to maintain normal function.

### Indirect functional consequences associated with the down-regulation of *Grxcr1*

A functional MET current during pre-hearing stages of development, which contributes to setting the resting membrane potential of hair cells, is essential for establishing the biophysical and morphological properties of mature IHCs [53]. The development of OHCs also appear to be highly dependent on them having normal biophysical characteristics during immature stages [57, 58]. In Tasmanian devil mice, the stereociliary bundles of hair cells are abnormally thin from around birth and become progressively more disorganized with age (Fig 1; [14]). The morphological defects in the stereociliary bundle of *tde/tde* mice caused a largely reduced size of the MET current and of the resting open probability (*Po*) of the MET channels. While the smaller MET current could be caused by the loss of tip links between the disorganized stereocilia, the reduced resting *Po* and its Ca^2+^ sensitivity are most likely caused by a reduced tension in the remaining tip links. A reduced MET channel *Po* has also been reported in several mouse models affecting the bundle structure (e.g. *Eps8* [37]; *Mir96* [59]) or Ca^2+^ handling at the stereocilia (*Atp2b2* [60]). The indirect functional consequence of the abnormal MET current apparatus was that Tasmanian devil mutant mice fail to acquire the adult-like basolateral membrane proteins, preventing them from becoming fully functional sensory receptors. This hypothesis is also supported by the observation of a similar failure in the acquisition of the mature IHC and OHC biophysical characteristics in mice lacking other stereociliary proteins associated with severe morphological hair bundle defects, such as TMC1 [61], EPS8 [37], USH1C and MYO7A [53].

### Role of Grxcr1 in hair bundle growth

Gene expression and immunostaining assays have shown that GRXCR1 is present in the sensory hair cells of the inner ear from early stages of development onwards, and localizes in the stereociliary bundle [10]. Cochlear hair cells from mice homozygous for mutations in the *Grxcr1* gene (pirouette and Tasmanian devil) have abnormally thin and long stereocilia, and their hair bundles become progressively more disorganized with age [10, 14]. Similar stereocilia defects were also seen in inner ear hair cells from *Grxcr1* zebrafish mutants [16]. Despite these abnormalities, the hair bundles establish their usual orientation (Fig 1B) and a rudimental staircase structure [2, 14], indicating that GRXCR1 is unlikely to influence the initial bundle morphogenesis. Instead, GRXCR1 appears to be primarily involved in regulating the widening and the final elongation of stereocilia, which in mammals occur simultaneously during the final stages of hair bundle formation [62]. Changes in the stereocilia dimensions are thought to involve several actin-bundling and -capping proteins together with the unconventional myosin motors and their cargo [1, 63].

The widening of the stereocilia is determined by the addition of actin filaments to the parallel actin bundle [2, 64], a process that depends on the ability of the actin-bundling protein ESPN to bind to and cross-link the actin filaments [17, 65]. ESPN is also involved in the elongation of the stereocilia since mice deficient for the protein show both abnormally thin and short stereocilia [17, 32, 66]. ESPN seems to require MYO7A and MYO3B in order for it to be transported to the stereocilia tip so it can exert its role in stereocilia elongation [67, 68]. In addition to MYO3 and ESPN, several other protein complexes are needed to achieve the extremely precise regulation of the stereocilia actin-cytoskeleton required to establish the fine structure of the hair bundle, with stereocilia length being identical among rows, and between neighbouring hair cells, but changing along the cochlea and between rows within each bundle [62]. The deafness gene *Eps8* (mice: [37]; human: [69]) encodes an actin-binding protein with multiple functions in the control of actin dynamics [70, 71]. In cochlear hair cells it is primarily localized at the tip of the longest row of stereocilia [18, 37, 72], and is key for their normal elongation by forming a complex with MYO15 and WHRN [18]. Another important protein complex is that formed by the unconventional MYO7A and its cargo TWF2, which is an actin capping protein able to restrict the elongation of the shorter rows of stereocilia [20, 73]. Stereocilia length can also be regulated by activity at the minus end of actin filaments at the base of the stereocilia [74]. PTPRQ is a receptor-like inositol lipid phosphatase required for normal stereocilia development [21], which requires the presence of MYO6 in order to localize to the stereocilia base [39]. Mutations in *Ptprq* lead to different degrees of hair bundle disorganization, including bundle shortening or giant stereocilia [21, 59].

In GRXCR1 deficient mice, we found ESPN, MYO7A, EPS8 and PTPRQ, which are involved in the four complexes listed above, surprisingly were distributed in a generally normal way within the mutant stereocilia despite the abnormalities visible in the hair bundles. GRXCR1 is an enzyme that has been shown to remove glutathione groups from target proteins, and it has been suggested to destabilize the interaction between the Usher Syndrome proteins USH1C and USH1G in zebrafish [16]. Although mice deficient for USH1C and USH1G do not exhibit thinner stereocilia, their hair bundles are profoundly disorganized [75]. Overall, our data indicate that GRXCR1 directly contributes to coordinating the final stages of hair bundle morphogenesis. This view is also supported by the observation that mice deficient for the paralogous vertebrate gene *Grxcr2*, which is also localized to the hair cell stereocilia, have profound hearing loss due to severe defects in the organization of their hair bundles [76].

## Supporting information

S1 File Novel transcript sequence of *Grxcr1*. 5’RACE PCR sequence of *Grxcr1* from exon 1 (normal type) to exon 4 (bold), including the entirety of exon 3 (underlined), and lacking exon 2.
(DOCX)
