# Supplementary material for: Grxcr1 regulates hair bundle morphogenesis and is required for normal mechanoelectrical transduction in mouse cochlear hair cells
Source: PLoS One. 2022 Mar 2;17(3):e0261530. doi: 10.1371/journal.pone.0261530 (PMC8890737; doi:10.1371/journal.pone.0261530)
Supplement: S1 File — 5’RACE PCR sequence of Grxcr1 from exon 1 (normal type) to exon 4 (bold), including the entirety of exon 3 (underlined), and lacking exon 2. (DOCX) [file pone.0261530.s001.docx]

tccggtttcg aattgcctca tctcacagtg ggagagttct gaaagaggtc tatgaagatg gacaagcacc aggctctctg gattctgaat gtgctagtat ttgtgccata gatgggctaa gtgactctga gggacagcaa aacggccaca ttggatcaga ggataatgaa caggagaaag atcaggataa cctgctggta ttagccagga cagccagcga gaaggctttt ggcacaagaa gagtcaacat tttaagcaaa aatggtacag tcagaggcgt caagtacaaa gtgagtgctg gccaggctct gtttaacaat ttgaccaaag tgttgcaggg tgctgagaaa attttgtcaa tgaatgaatc aggagaactg caagaccttt taaccaaaat tgag**agggtc cagcatccgc acgagtgtcc ttcctgtgga ggctttggct tcc**

**S1 Sequence file. Novel transcript sequence of *Grxcr1*.** 5’RACE PCR sequence of *Grxcr1* from exon 1 (normal type) to exon 4 (bold), including the entirety of exon 3 (underlined), and lacking exon 2.
